# A causal analysis of school exclusions and youth custody using administrative data and a bit of luck

**DOI:** 10.23889/ijpds.v8i2.2339

**Published:** 2023-09-14

**Authors:** Alex Sutherland, Giulia Tagliaferri

**Affiliations:** 1 Research Design UK, Cambridge, United Kingdom; 2 Department of Social Policy & Intervention, University of Oxford, Oxford, United Kingdom; 3 Behavioural Insights Team, London, United Kingdom

## Objectives

There is a strong relationship between being excluded from school and experiences of criminality and custody in later life. But we don’t know if this relationship is causal: does being excluded cause more people to commit crime and end up in custody? This study tries to answer this question directly.

## Methods

We linked data from the National Pupil Database and the National Client Caseload Information System for four cohorts of Yr10 pupils attending state-funded schools in England.

We analysed the effect of being permanently excluded or suspended in Year 10 on the probability of custody at age 15-17 (inclusive) for more than a million pupils. We used academisation, when schools move out of local council control to greater self-governance, to understand the impact of exclusions on custody. We treat academisation as a ‘shock’ that increases the likelihood of exclusion then use a statistical approach called instrumental variables that enables us to leverage this change to understand the impact on later custody.

## Results

Attending a school that converts to an academy in Year 10 increases the probability of a pupil receiving a suspension or permanent exclusion by 3 percentage points. A Year 10 pupil attending a school that academised resulted in a statistically significant increase in the probability of custody age 15-17, with impacts varying depending on the type of exclusion:

- a permanent exclusion increases the probability of custody by 33 percentage points (statistically significant at 5%);
- for suspension, the increase is 1.3 percentage points (statistically significant at 5%)

## Conclusion

Employing a novel dataset combined with an approach that allows us to distinguish causal relationships, we can make stronger claims about the impact of exclusion on custody (and thus crime). Our results highlight an onward negative criminal justice outcome of education policy that, to the best of our knowledge, has not previously been quantified. Exclusion presents a small but non-ignorable risk of increases in custody, warranting greater scrutiny.

